# Supplementary figures and images for: Loss of CITED1, an MITF regulator, drives a phenotype switch in vitro and can predict clinical outcome in primary melanoma tumours
Source: PeerJ. 2015 Feb 26;3:e788. doi: 10.7717/peerj.788 (PMC4349148; doi:10.7717/peerj.788)

**a**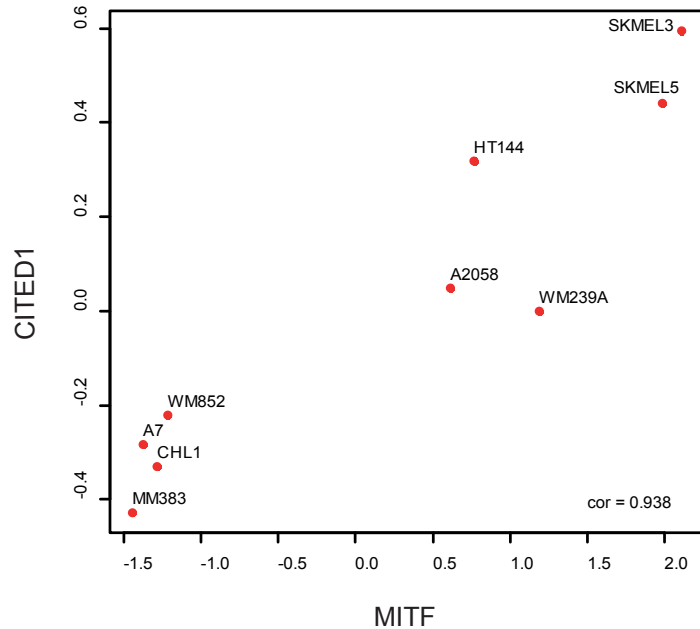**b**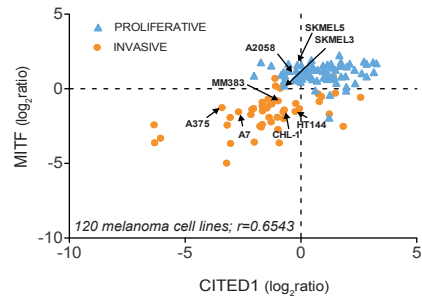

Supplement: Figure S1 — (A) A scatter plot of CITED1 expression versus MITF expression in our in-house cell lines subjected to gene expression analysis in agreement with the observation in the public data set, (see Fig. 2A. (B) Fig. 2A reproduced) with additional labelling to indicate the common cell lines. [file peerj-03-788-s001.pdf]

**a**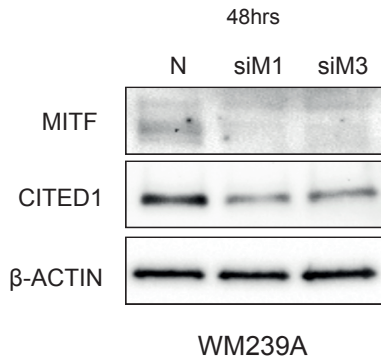**b**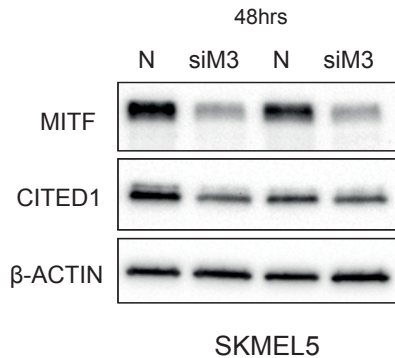

Supplement: Figure S3 — (A) Western blot showing the effect of silencing MITF using two siRNAs (siM1, siM3) on both MITF and CITED1 levels in WM239A cells relative to a negative control siRNA (N) at 48 h post-transfection. β-Actin is used as a loading control. (B) Western blot showing the effect of silencing MITF using the siRNA siM3 on both MITF and CITED1 levels in SKMEL5 cells relative to a negative control siRNA (N) at 48 h post-transfection. β-Actin is used as a loading control. [file peerj-03-788-s003.pdf]

**a**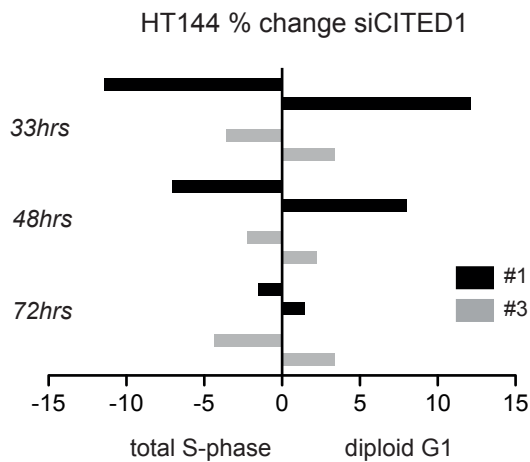**b**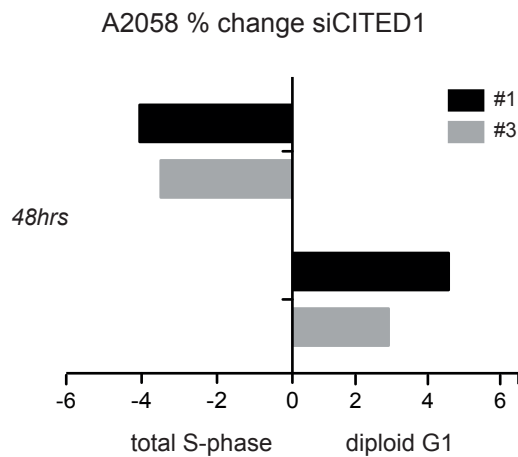**c**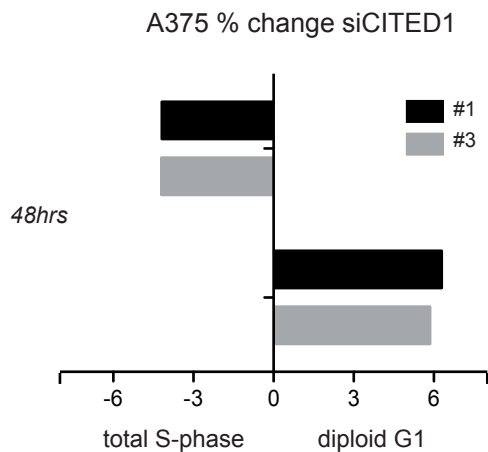

Supplement: Figure S4 — (A) A bar chart showing the % change in cell cycle distribution in both #1 and #3 siCITED1 treated HT144 cells relative to siNEG treated HT144 cells. The reduction in total S-phase is shown at 33 h, 48 h and 72 h post-transfection in addition to the corresponding increase in the diploid G1 fraction. (B) A bar chart showing the % change in cell cycle distribution in both #1 and #3 siCITED1 treated relative to siNEG treated A2058 cells. The reduction in total S-phase and corresponding increase in G1 is shown at 48 h post-transfection. (C) A bar chart showing the % change in cell cycle distribution in both #1 and #3 siCITED1 treated relative to siNEG treated A375 cells. The reduction in total S-phase and corresponding increase in G1 is shown at 48 h post-transfection. [file peerj-03-788-s004.pdf]

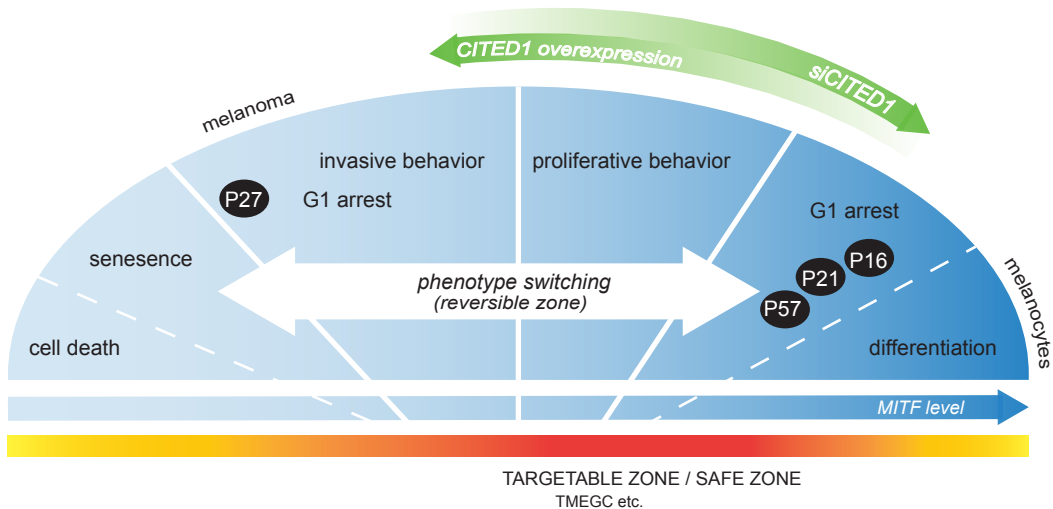

Supplement: Figure S6 — The rheostat model of MITF action in melanoma indicating the hypothesised role of CITED1 in the regulation of MITF expression and melanoma cell behaviour. The model is an original adaptation of those previously proposed by others, now incorporating our data (Hoek & Goding, 2010; Carreira et al., 2006; Cheli et al., 2011a; Cheli et al., 2011b). [file peerj-03-788-s006.pdf]
