# Supplementary material for: Loss of CITED1, an MITF regulator, drives a phenotype switch in vitro and can predict clinical outcome in primary melanoma tumours
Source: PeerJ. 2015 Feb 26;3:e788. doi: 10.7717/peerj.788 (PMC4349148; doi:10.7717/peerj.788)
Supplement: Figure S2 — (A) Of the 5578 unique occupied genes (those associated with genomic regions bound by MITF) identified by Strub et al., 5169 could be found in the Illumina HT12 gene set, mapping to 8272 probes by searching on gene symbol (Strub et al., 2011). Of the 312 probes significantly changed by siCITED1, 41% or 128 are found in this list. (B) A Venn diagram indicating the number of genes significantly up or down regulated by siCITED1 represented in the MITF occupied gene list. [file peerj-03-788-s002.pdf]

a

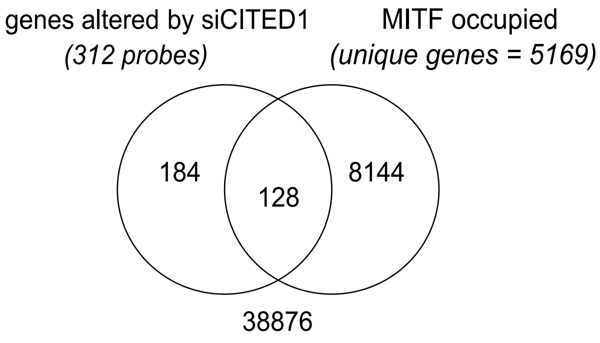

Chi-sq (yates) p-value < 0.0001

b

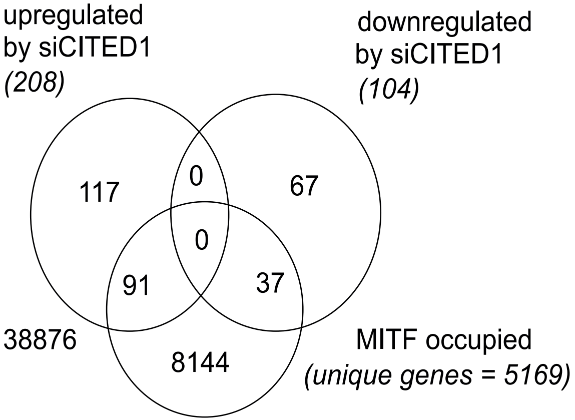

Illumina probes

	MITF occupied	Not occupied	Total
sig changed with siCITED1	128	184	312
not signif	8144	38876	47020
Total	8272	39060	47332
