# Supplementary material for: Loss of CITED1, an MITF regulator, drives a phenotype switch in vitro and can predict clinical outcome in primary melanoma tumours
Source: PeerJ. 2015 Feb 26;3:e788. doi: 10.7717/peerj.788 (PMC4349148; doi:10.7717/peerj.788)
Supplement: Figure S5 — Western blot of melanoma cells lines indicating the differential expression of the cell cycle regulator CDKN1A/P21 and CDKN1C/P57 in addition to MITF and CITED1. β-Actin is used as a loading control. [file peerj-03-788-s005.pdf]

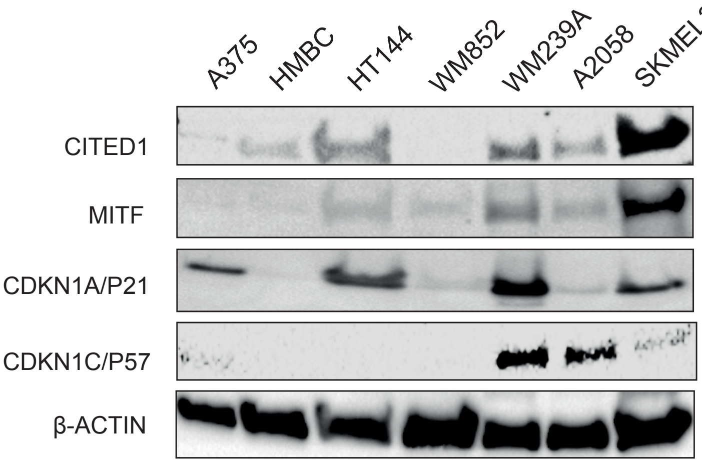
A375
HMBC
HT144
WM852
WM239A
A2058
SKMEL3
CITED1
MITF
CDKN1A/P21
CDKN1C/P57
β-ACTIN
